# Floristic Inventory and Diversity Assessment at Two Locations along the Shores of Cape Coast, Ghana

**DOI:** 10.1155/2024/5195390

**Published:** 2024-08-29

**Authors:** Gertrude Lucky Aku Dali, Sethiler Arthur, Paul Kwame Essandoh

**Affiliations:** Department of Environmental Science School of Biological Sciences University of Cape Coast, Cape Coast, Ghana

## Abstract

Coastal vegetation plays significant roles such as stabilization of the surface against wind and erosion, and provision of critical terrestrial and aquatic habitats for organisms. Floristic studies serve as a way of monitoring and evaluating the health of ecosystems. Currently, information on the floristic composition and diversity along the shoreline of Cape Coast is scanty. The study was aimed at assessing the impacts of anthropogenic activities on plant biodiversity along the shoreline of Cape Coast, Ghana. Thus, the study analysed the biodiversity of plants at the Hutchland beach (a disturbed area) and the Asasse Pa beach (a fairly undisturbed area). It was hypothesised that the Asasse Pa beach had a higher species diversity than the Hutchland beach. An inventory was made of all plant species found at both locations. A belt transect method was used for the ecological study, involving the use of a 1 m^2^ quadrat. All the plants that were found in each quadrat were identified, and the species and number of individuals of each species were counted—this information was used in the determination of the ecological parameters of the species and the locations. Parameters between the two locations were compared with a *t*-test, whereas variations in the distribution of the species were determined with principal components analysis (PCA), using Minitab and R statistical software, respectively. A total of 50 plant species belonging to 48 genera and 23 families were inventoried along the shoreline. The family Poaceae had the highest number of species, 10. The Asasse Pa beach had a higher species diversity and evenness of 2.84 and 0.84, respectively, whereas the Hutchland beach had a lower species diversity and evenness of 2.44 and 0.75, respectively. Anthropogenic activities at the Hutchland beach might have accounted for the lower species diversity and evenness there. The study therefore recommends periodic monitoring of coastal vegetation also law enforcement on coastal resources.

## 1. Introduction

Coastal ecosystems consist of coastal lands and nearshore marine areas, which are highly linked, with water serving as the enabler of almost all the linkages [1]. They are interfaces where the sea meets the land encompassing shoreline environments [2]. They may be broadly defined as the part of the sea influenced by the land and the part of the land that is influenced by the sea [3]. Some of the major habitats in the coastal ecosystems include wetlands, estuaries, coastal lagoons, mangrove forests, salt marshes, tidal areas, intertidal flats, rocky shores, coastal plains, reefs, and sandy beaches.

Globally, coastal ecosystems are found in 123 countries [4], including Ghana. Ghana has a coastline of 550 km [5]. Coastal areas serve as homes to billions of people who directly or indirectly use coastal ecosystems as their sources of livelihood [6]. There is continual migration of people from inland areas to the coastal areas [7]. Therefore, coastal populations are said to be increasing rapidly [1, 8]. It was projected that two-thirds of the world's population lives either in or around the coast [9]. An increase in coastal populations puts immense pressure on coastal vegetation.

The coastal inhabitants depend heavily on coastal ecosystems and their services such as storm buffering and fisheries, as well as homes to a wide range of biodiversity. For instance, plants or vegetation including mangroves and coconut trees along the coastline provide ecological services such as primary production, carbon fixation, and water filtering [10]. They also protect shorelines and hold back sediments from the effects of erosive processes including tides, waves, and storms. In addition, they play a significant role in stabilizing the surface against wind and erosion. Without vegetation, the transportable strata of coastal land erode and the surrounding area loses the safety of the coastal area [11].

Despite the enormous importance of coastal ecosystems, they are being destroyed and lost at alarming rates [12]. The global rate of coastal ecosystem loss was estimated as follows: mangroves, 1–3% [13]; seagrass meadows, 2–5% [14]; and corals, 4–9% [15]. These losses erode biodiversity, as well as the valuable ecosystem services they provide [16]. Coastal vegetation, for example, is being degraded due to human activities such as developmental projects going on along the shores. A study conducted in Ghana reported 11.79% and 15.05% reduction in coastal vegetation cover for the periods, 1996–2017 and 1996–2002, respectively [17]. Modifying the coastal environment to meet the needs of the society is causing severe effects, such as global warming, ecological collapse, mass extinction, and biodiversity loss [18]. The human-caused decline in plant biodiversity has dire consequences for the well-being of human beings themselves [19], as well as other organisms. Consequently, human influence on coastal ecosystems is considered one of the dangerous environmental threats of our time [6]. Furthermore, the Anthropocene is highly linked to the rapid loss of species and the vanishing of their major ecosystem functions [20]. Anthropocene is a term coined by Crutzen and Stoermer in 2000 to highlight that human impacts on the environment are as important as, or more important, than the impacts of natural forces or processes on the environment [21]. It thus refers to how human activities greatly altered conditions and processes on the entire Earth. The rate at which coastal vegetation is being degraded calls for a lot of attention. Hence, it is very necessary to monitor and put data together, to help improve the vegetation along the coasts. This study is very crucial since the outcome will provide baseline information necessary for monitoring the shoreline. This is because monitoring is one of the ways of protecting the shoreline and preventing it from rapid depletion, for the maintenance of its maximum benefits.

Undeniably, studies have been carried out around the world including Ghana on coastal vegetation. For instance, vegetation analysis and floristic of four communities were conducted in the Big Ball Hill region of Padre Island National Seashore [22]. The composition, structure, and diversity of coastal vegetation in the northeastern part of Cozumel in Mexico were also studied [23]. In Ghana, however, most of the studies on coastal vegetation generally focused on wetlands and mangrove ecosystems [12, 24–30]. Data on vegetation along the shores of Cape Coast are lacking. Therefore, this study sought to provide data on vegetation at two locations along the coast of Cape Coast, which on a whole, will help to conserve vegetation along the coast of Ghana. The study was undertaken with the following specific objectives: (1) to make an inventory of plants (herbs and saplings) along the shoreline of Cape Coast, (2) to determine the ecological parameters of plant species, and (3) to assess the impacts of human activities on plant biodiversity. It was hypothesised that the fairly undisturbed area (Asasse Pa beach) had a higher species diversity than the disturbed area (Hutchland beach). The outcome of this study would serve as baseline information on vegetation along the shoreline of Cape Coast and would also help reduce human activities along the shorelines, towards the conservation of biodiversity. Findings from the study may also contribute to the sustainable development goals (SDGs) in line with life on land (goal 15), life below water (goal 14), climate action (goal 13), sustainable communities (goal 11), and poverty alleviation (goal 1). Goal 15 is to halt and reverse land degradation and halt biodiversity loss; goal 14 is to conserve and sustainably use marine resources for sustainable development; goal 13 is to combat climate change and its impacts; goal 11 is to make communities safe, resilient, and sustainable; and goal 1 is to end poverty in all its forms everywhere. This research would, therefore, help address aspects of these goals.

## 2. Materials and Methods

### 2.1. Study Area

**Figure 1:**
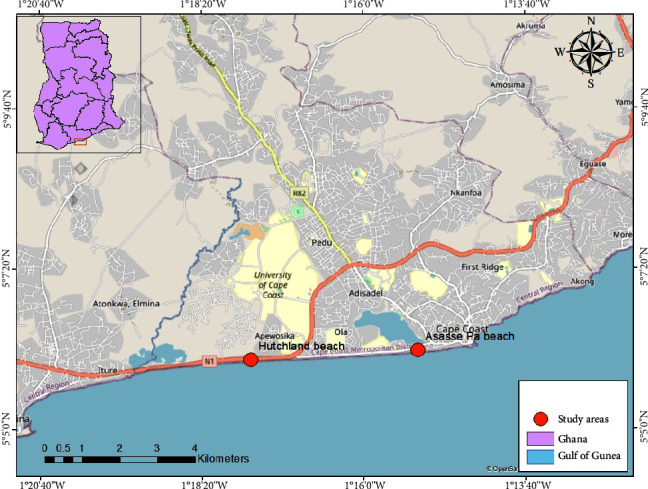
Map showing Hutchland and Asasse Pa beaches.

The study was carried out at two different locations, Hutchland and Asasse Pa beaches along the shoreline in Cape Coast (Figure 1). The Hutchland beach is a resort centre located along Cape Coast-Takoradi trunk road, about 4.2 km away from the University of Cape Coast. It lies between latitude 5°06′00.2″ N and longitude 1°17′38.2″ W, Cape Coast. Asasse Pa beach is an abandoned resort centre located in Bakano, close to the Fosu Lagoon, and about 6.1 km away from the University of Cape Coast and lies between latitude 5°06′08.2″N and longitude 1°15′13.6″W.

Along the shore of Cape Coast are a number of recreational and tourist facilities such as beach resorts and restaurants. The Cape Coast catchment experiences two peaks of wet season. The major peak occurs from May to July, whereas the minor peak occurs from September to December. Two dry seasons occur; the short one in August, and the long one from January to April, with February and March normally being the hottest months. Cape Coast is a humid area with mean monthly relative humidity varying between 85% and 99%. The soil type is mostly sandy soil, however, particles of mud intermittently mix with the sandy soil caused by wave actions that continually move these particles ashore [31]. Some plant species found along the stretch of the coastline are coconut trees (*Cocos nucifera*), *Opuntia* sp. *Thespesia populnea*, and *Imperata cylindrica*. However, detailed information on the plants along the shoreline of Cape Coast is very scanty.

### 2.2. Methods

#### 2.2.1. Inventory and Sampling of Plant Species

**Figure 2:**
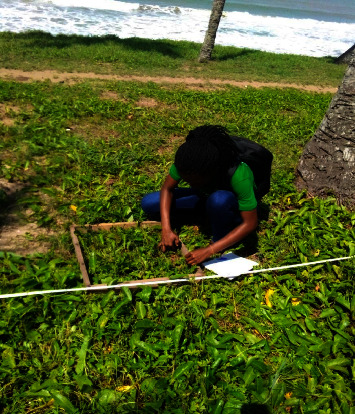
Sampling of plant species.

Plant biodiversity along the shoreline of Cape Coast was assessed through botanical inventories, transects, and quadrats. Botanical inventories involved walking through different locations at the Hutchland (disturbed area) and Asasse Pa (fairly undisturbed area) beaches and picking and identifying the plant species [32]. A belt transect method was used to sample and quantify herbaceous plant species and saplings. Herbaceous plants are plants that have flexible, green, and nonwoody stems. Saplings are young trees with tender trunks. A transect line of 100 m was laid out along the shores at both study sites, using a surveyor's tape. A 1 m^2^ quadrat was placed on the first marked point on the line, and later at 10 m intervals, making a total of 10 quadrats per study location (Figure 2). All the plant species comprising herbaceous plants and saplings that were found within each of the quadrats were identified, counted, and recorded. The number of individuals of each species was also counted. Species that could not be identified on the field were labeled and counted and sent to the School of Biological Sciences Herbarium for identification, using manuals [33–36]. After the identification, all the species were classified into their taxonomic families using appropriate manuals. Each of the identified plant species was also categorized into growth forms or habits.

#### 2.2.2. Data Analyses

The ecological parameters were determined for each of the species and the study locations as follows:

(1) $$\begin{array}{c} \text{Density}=\frac{\text{Total number of individuals of }a\text{ species}}{\text{Total area studied}},\\ \text{Frequency}=\frac{\text{Number of quadrat in which }a\text{ species occurs}}{\text{Total number of quadrat studied}}\times 100\%,\\ \text{Abundance}=\frac{\text{Total number of individuals of }a\text{ species present in all quadrats }}{\text{Number of quadrats of occurrence of the species}},\\ \text{Relative Density }\left(\text{RD}\right)=\frac{\text{Individual density of }a\text{ species}}{\text{Total density of all species}}\times 100\%,\\ \text{Relative frequency }\left(\text{RF}\right)=\frac{\text{Individual }f\text{requency of }a\text{ species}}{\text{Total frequency of all species}}\times 100\%,\\ \text{Relative abundance }\left(\text{RA}\right)=\frac{\text{Individual abundance of }a\text{ species}}{\text{Total abundance of all species}}\times 100\%,\\ \text{Importance Value Index }\left(\text{IVI}\right)=\sum \left(\text{RD}+\text{RF}+\text{RA}\right),\\ \text{Composition}=\frac{\text{Number of individuals of }a\text{ species}}{\text{Total number of individuals of all species}}\times 100\%,\\ \text{Shannon}-\text{Weaver diversity index }\left(H^{\prime }\right)=-\sum \rho i\text{ In }\rho i,\\ \text{Evenness}=\frac{H^{\prime }}{\text{In}\left(S\right)}, \end{array}$$

where *H*′ is the Shannon–Weaver diversity index.

(2) $$\begin{array}{c} \text{Similarity index }\beta =\frac{2C}{S1+S2}, \end{array}$$

where pi = ni/*N*, ni is the number of individuals of the *i*^th^ species, *N* is the total number of individuals, *S* is the number of species, *S*1 is the number of species in community 1, *S*2 is the number of species in community 2, and *C* = number of species common to both communities.

Dominance and abundance are two interchangeable characteristics that are mostly used together with density and frequency in the estimation of the Importance Value Index (IVI) in the studies of plant dynamics [37]. Since this study focused on herbaceous plants and saplings, abundance was used in estimating the IVI.

Species diversity and species evenness were calculated for each quadrat after which a Student's *t*-test was done to compare species diversity and species evenness between the two locations using, Minitab software. In addition, principal components analysis (PCA) was performed using R statistical software to show the variations in the distribution of the species along the coastline.

## 3. Results

### 3.1. Inventory of Plant Species along the Shoreline

A total of 50 plant species were found along the shoreline specifically, at the Hutchland and Asasse Pa beaches (Table 1). These species belonged to 48 genera and 23 families. Poaceae had the highest number of species, 10, followed by Amaranthaceae with five species. Families including Acanthaceae, Aizoaceae, Arecaceae, Cactaceae, Combretaceae, Cucurbitaceae, Euphorbiaceae, Nyctaginaceae, Passifloraceae, Phyllanthaceae, and Zygophyllaceae recorded only one species each. A majority of the plant species (86%) were herbs, while 8% and 6% of them were saplings of shrubs and trees, respectively.

### 3.2. Ecological Importance of Plants at the Hutchland Beach

**Figure 3:**
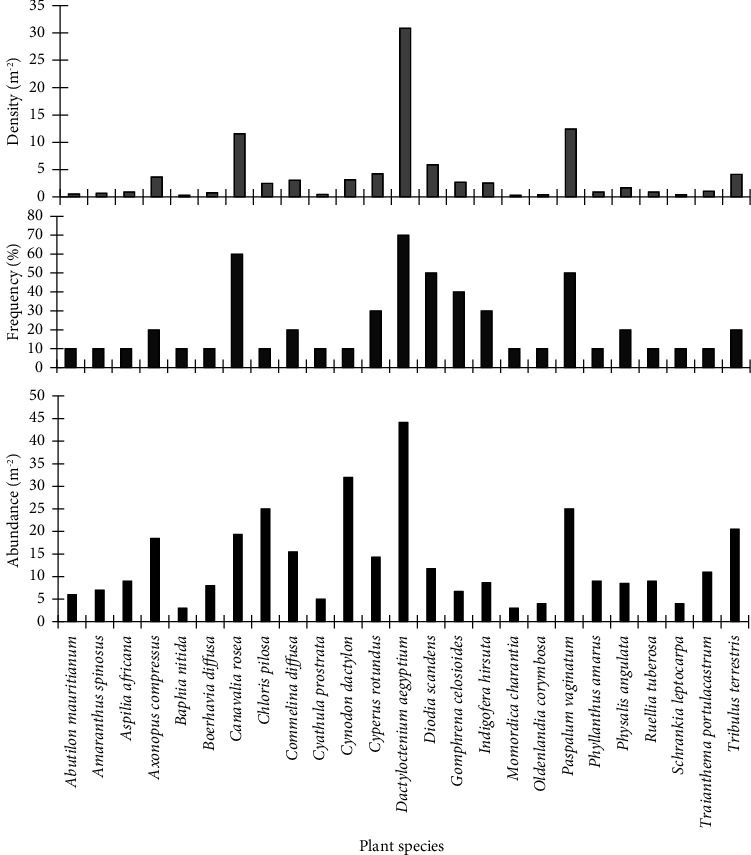
Ecological parameters of plants at the Hutchland beach.

A total of 25 plant species were found within the quadrats laid at the Hutchland beach. *Dactyloctenium aegyptium* had the highest ecological parameters (Figure 3). Its density, frequency, and abundance were 30.9 m^−2^, 70%, and 44.1 m^−2^, respectively. Other species that had high ecological parameters included *Paspalum vaginatum*, *Canavalia rosea*, and *Diodia scandens*. Species that had the lowest ecological parameters included *Momordica charantia*, *Baphia nitida*, and *Schrankia leptocarpa*.

**Figure 4:**
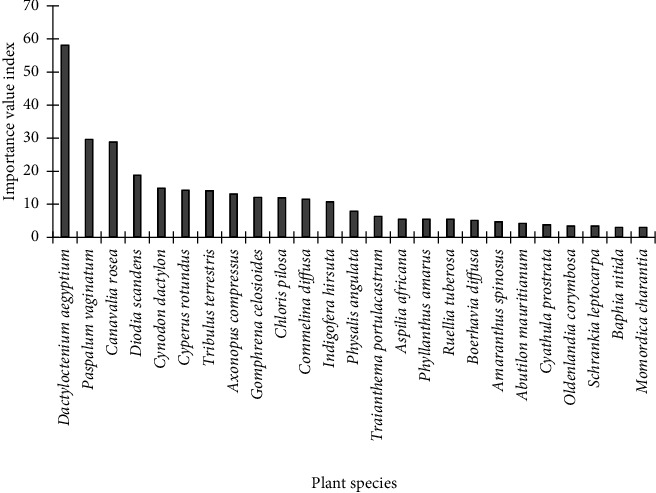
Importance value indices of plants at the Hutchland beach.

*D. aegyptium* was the topmost ecologically important species, about 50% more than *P. vaginatum*, the second most ecologically important species at the Hutchland beach (Figure 4). While *D. aegyptium* had IVI of 58.2, that of *P. vaginatum*, was 29.7. *C. rosea*, and *D. scandens* were the next most ecologically important species with IVI of 28.8 and 18.8, respectively. The least ecologically important species were *M. charantia* and *B. nitida*, with IVI of 3.0 each.

### 3.3. Ecological Importance of Plants at the Asasse Pa Beach

**Figure 5:**
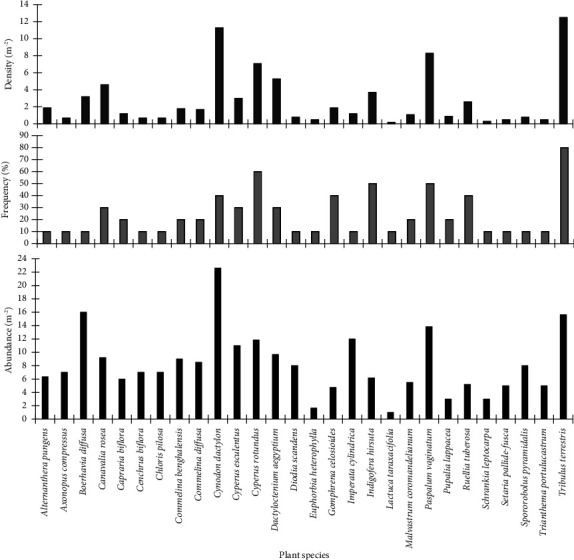
Ecological parameters of plants at the Asasse Pa beach.

A total of 29 plant species were sampled at the Asasse Pa beach. *Tribulus terrestris* and *Lactuca taraxacifolia* were the species with the highest and lowest ecological parameters, respectively (Figure 5). *T. terrestris* recorded density, frequency, and abundance of 12.5 m^−2^, 80%, and 15.63 m^−2^, respectively. *L. laraxalifolia* had density, frequency, and abundance of 0.2 m^−2^, 10%, and 2.0 m^−2^, respectively.

**Figure 6:**
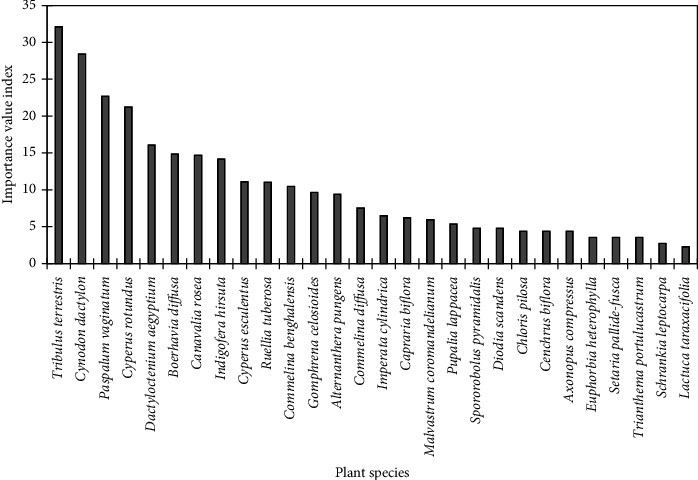
Importance value indices of plants at the Asasse Pa beach.

Overall, species of ecological importance at the Asasse Pa beach were *T. terrestris*, *Cynodon dactylon*, *D. aegyptium*, *P. vaginatum*, and *Cyperus rotundus*, with IVI of 32.16, 28.45, 26.63, 22.72, and 21.27, respectively (Figure 6). *L*. *taraxacifolia* and *Schrankia leptocarpa* were the species with the least ecological importance, with IVI of 2.31 and 2.73, respectively.

### 3.4. Ecological Parameters of Plant Species along the Coastline

The overall ecological parameters of plants along the shore are shown in Table 2. The species with the highest ecological parameters comprising IVI and composition of 19.1 and 35.4 5%, respectively, was *Dactyloctenium aegyptium*. It was about twice more ecologically important than other ecologically important species.

**Figure 7:**
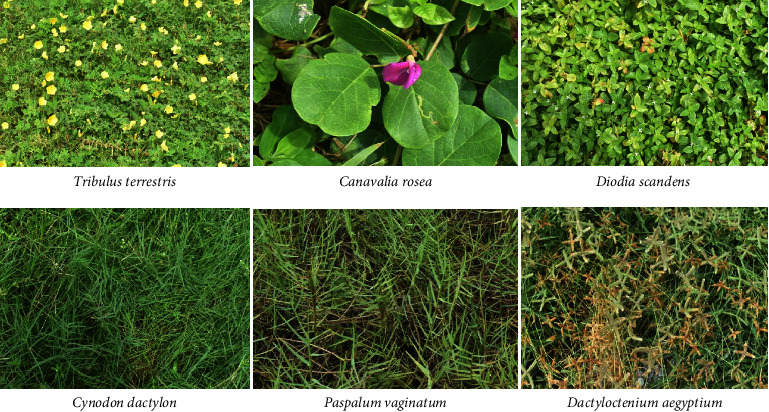
Images of some of the most ecologically important plant species.

*Paspalum vaginatum*, *Tribulus terrestris*, and *Cynodon dactylon* were the next species of high IVI and compositions of 11.4 and 24.3%, 10.2 and 22.9%, and 9.06 and 20.5%, respectively. The species with the lowest IVI (1.49) and composition (0.13%) was *Lactuca taraxacifolia*. Images of some of the most ecologically important plant species are shown in Figure 7.

### 3.5. Ecological Community Parameters for the Asasse Pa and Hutchland Beaches

A total number of plants recorded at the Asasse Pa and Hutchland beaches were 29 and 25, respectively (Table 3). The species diversity and evenness were 2.84 and 0.84, and 2.44 and 0.75 for the Asasse Pa and Hutchland beaches, respectively. The difference in the species diversity of the two locations was significant (*t* = −6.34, *p* < 0.05), while that of species evenness was nonsignificant (*t* = −1.74, *p* > 0.05). The Similarity Index for Asasse Pa and Hutchland beaches was 0.54.

### 3.6. Principal Component Analysis of the Distribution of Plant Species along the Coastline

The PCA of the distribution of the plant species at each location yielded nine components or dimensions each. Five components accounted for 84.5% and 73.3% of the variations at the Hutchland and Asasse Pa beaches, respectively (Table 4). Component one (Dim.1) accounted for 22.5% and 19.7%, while component two (Dim.2) accounted for 20.3% and 15.7 5% of the variations at the Hutchland and Asasse Pa beaches, respectively.

Six species that contributed to the principal components at the Hutchland beach were *Canavalia rosea*, *Indigofera hirsuta*, *Diodia scandens*, *Boerhavia diffusa*, *Paspalum vaginatum*, and *Cyperus rotundus* (Table 5).

**Figure 8:**
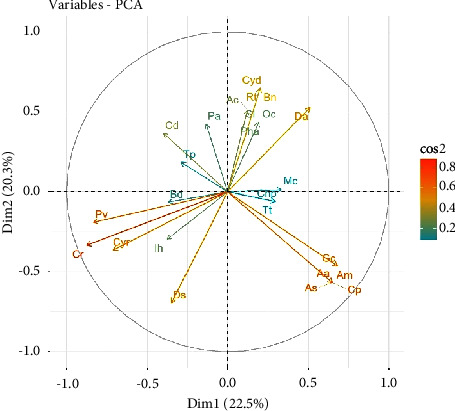
Correlation circle showing the covariates in a two-dimensional plot (Dim.1 × Dim.2) at the Hutchland beach. Cr = *Canavalia rosea*, Ih = *Indigofera hirsuta*, Ds = *Diodia scandens*, Bd = *Boerhavia diffusa*, Pv = *Paspalum vaginatum*, Cyr = *Cyperus rotundus*, Gc = *Gomphrena celosioides*, Ac = *Axonopus compressus*, Da = *Dactyloctenium aegyptium*, Tt = *Tribulus terrestris*, Cd = *Commelina diffusa*, Pa = *Physalis angulata*, Tp = *Trianthema portulacastrum*, Rt = *Ruellia tuberosa*, Cyd = *Cynodon dactylon*, Bn = *Baphia nitida*, As = *Amaranthus spinosus*, Cp = *Cyathula prostrata*, Am = *Abutilon mauritianum*, Aa = *Aspilia africana*, Oc = *Oldenlandia corymbosa*, Sl = *Schrankia leptocarpa*, Pha = *Phyllanthus amarus*, Mc = *Momordica charantia*, Chp = *Chloris pilosa*.

Dim. 1 and Dim. 2 accounted for 22.5% and 20.3% of the variations in the distribution of plant species at the Hutchland beach (Figure 8). The species that contributed most to the variations were *Canavalia rosea, Cyathula prostrata, Amaranthus spinosus*, *Paspalum vaginatum*, and *Diodia scandens*.

Six species that contributed to the principal components at the Asasse Pa beach were *Tribulus terrestris*, *Ruellia tuberosa*, *Canavalia rosea*, *Paspalum vaginatum*, *Euphorbia heterophylla*, and *Alternanthera pungens* (Table 6).

**Figure 9:**
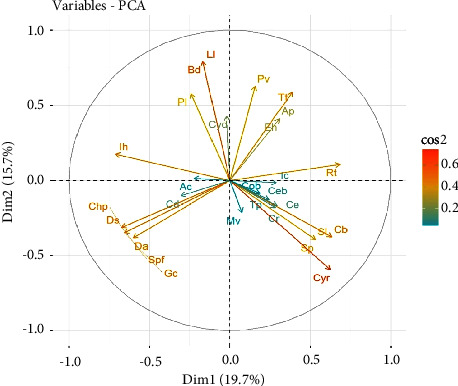
Correlation circle showing the covariates in a two-dimensional plot (Dim.1 × Dim.2) at the Asasse Pa beach. Tt = *Tribulus terrestris*, Rt = *Ruellia tuberosa*, Cr = *Canavalia rosea*, Pv = *Paspalum vaginatum*, Eh = *Euphorbia heterophylla*, Ap = *Alternanthera pungens*, Ih = *Indigofera hirsuta*, Cyd = *Cynodon dactylon*, Bd = *Boerhavia diffusa*, Pl = *Pupalia lappacea*, Ll = *Lactuca taraxacifolia*, Sp = *Spororobolus pyramidalis*, Sl = *Schrankia leptocarpa*, Cb = *Capraria biflora*, Cyr = *Cyperus rotundus*, Gc = *Gomphrena celosioides*, Ds = *Diodia scandens*, Chp = *Chloris pilosa*, Spf = *Setaria pallide-fusca*, Da = *Dactyloctenium aegyptium*, Ceb = *Cenchrus biflorus*, Ce = *Cyperus esculentus*, Mv = *Malvastrum coromandelianum*, Tp = *Trianthema portulacastrum*, Ac = *Axonopus compressus*, Cd = *Commelina diffusa*, and Ic = *Imperata cylindrica*.

Dim. 1 and Dim. 2 accounted for 19.7% and 15.7% of the variations in the distribution of plant species at the Asasse Pa beach (Figure 9). The plant species that contributed most to the variations were *Cyperus rotundus*, *Lactuca taraxacifolia*, and *Boerhavia diffusa*.

## 4. Discussion

The goal of floristic or plant inventory is to produce a comprehensive list of plants found in a particular place at a particular time, to serve as a baseline for monitoring and evaluating future conditions. Floristic inventory is a general practice throughout the world to gather more information about plants in an area of interest [38]. Assessments of floristic composition and diversity are essential to understanding the present diversity status and conservation of plant biodiversity [39]. The total of 50 plant species encountered in this study is an indication that the shoreline of Cape Coast has different floristic compositions. This information is very vital in our attempts to protect our coastline because we can only protect our resource better, only when we know what it contains and how it functions. This is because floristic inventory provides important data for several purposes such as land management and biodiversity conservation. High coastal plant biodiversity is necessary for the maintenance of valuable coastal wetland ecosystem services such as flood regulation, sediment retention, nutrient cycling, and carbon sequestration.

Taxonomic information on species in an area provides an overview of the kinds of lineage and traits shared among the species. The Poaceae (grass or Gramineae) family recorded the highest number of species found in this study. This may be because of the ability of grasses to grow almost everywhere, throughout the globe [40]. Grasses are annual or perennial herbaceous, monocotyledonous plants with jointed stems (often hollow) with distinct nodes and internodes, and sheathed leaves. Poaceae is also the most economically important angiosperm family in the world [40, 41]. Considering the importance of Poaceae, the conservation of these species contributes not only to the ecological importance but also to the economic importance of the coastal environment.

*Dactyloctenium aegyptium*, family Poaceae, was the most ecologically important plant species found in the sampled areas along the shores of Cape Coast. This implied that it was frequently found in large numbers of individuals along the shores. This finding supports a recent study in India that reported *D. aegyptium* as one of the dominant grasses present in coastal habitats [42]. *D. aegyptium* is considered a pioneer grass species that swiftly occupies or colonises disturbed areas accumulated with water or close to the coasts [43]. This may explain why *D. aegyptium* dominated the Hutchland beach and was twice more ecologically important than the second ecologically important species. A study on alien and potentially invasive plants in lagoons in Mexico recorded the presence of *D. aegyptium* in three out of four lagoons [44], indicating how invasive this species can be.

*Paspalum vaginatum* was the next most ecologically important species, basically due to its high frequency and density. This implies that it was frequently present or common, as well as numerous along the shoreline. *P. vaginatum* is widely recognized as a serious invasive plant that rapidly transforms ecosystems and poses detrimental effects on those ecosystems [45]. *P. vaginatum* was found to be a serious threat to the ecological function and structure of wetlands in California [46]. Since there is inadequate data on the composition of species along the coast of Cape Coast, it is difficult to indicate whether *P. vaginatum* had changed the composition and structure of plants long the shoreline. However, its reportedly rapid invasive nature could have contributed to the smaller number of native seashore plants such as *Opuntia ficus-indica*, which did not occur in any of the quadrats thrown. Also, no sapling of *Cocos nucifera*, another typical coastline plant, was found within the quadrat throws. It is possible that the reduction in the number of *Cocos nucifera* along the coast also contributed to the high abundance of *P. vaginatum*. This is because it potentially outcompetes and inhibits germination of other delicate plants and displaces and eliminates recruitment of native species [46]. Adequate numbers of saplings are good indicators of successful regeneration ability of a vegetative community [47]. Tree regeneration generally refers to the process whereby a forest or plant community sustains itself through the growth and survival of seedlings and saplings that develop *in situ* [48]. Therefore, the absence or limited numbers of saplings in a plant community are indications of the presence of threatened trees. This implies that there is a high possibility that the native tree species might be prone to local extinction in the future, due to the absence of saplings of these trees [49].

Again, the density of *P. vaginatum* at the Hutchland beach (12.5 m^−2^) was higher than that at the Asasse Pa beach (8.3 m^−2^). This may also be due to the invasive nature of this species, making it thrive well in disturbed areas. This suggests that the species is stress-tolerant and can adapt to various anthropogenic activities [50]. Elsewhere, *P. vaginatum* was reported as the most abundant and possibly invasive plant species [44]. Biological invasion or invasive species often alter the composition of species on the ground flora, hence reducing plant diversity, and favouring exotic associations [51]. From the PCA, *P. vaginatum* was found to be among the six species that contributed to the principal components at the two beaches (Tables 5 and 6), an indication of its contributions to the variations in the species distribution along the shoreline. This may imply that *P. vaginatum* is an indicator species along the shoreline of Cape Coast.

*Tribulus terrestris* was also one of the most ecologically important species found at the shoreline. It had the highest frequency of occurrence along the shoreline (Table 2) and was the most ecologically important species at the Asasse Pa beach (Figure 5). It also contributed to principal components, thus, variations in the species distribution at the Asasse Pa beach (Table 6). *T*. *terrestris* has been widely known as an important medicinal plant for a very long time [52]. For instance, it is valued for its natural remedies, such as treatment for erectile dysfunction, stimulating the immune system [53], sexual disorders, infertility, chest pain, an enlarged prostate, and many other conditions. It could also have a potential for effective blood pressure control because of its diuretic, cardioprotective, and antihyperlipidemic activities [54]. Thus, it has a high and persistent use by the pharmaceutical industries which resulted in high production demands that raised the need for it to be semicultivated [52].

Although the pharmaceutical and international trade potentials were confirmed for *T*. *terrestris*, the plant was affected by changes in environmental factors [55]. Conservation and sustainable use of this species will be, therefore, beneficial to medicinal plant researchers and businesses [53], as well as herbal practitioners. This will in turn contribute to the prevention of loss of biodiversity and reduction in poverty, especially among the users of this plant and individuals along the coasts.

Floristic diversity reflects environmental conditions, physiognomy, and biotic influences of an area. Plant biodiversity indicates the number of different plant species found in a particular area, and it is often represented by species diversity. Species diversity is a standardized index that combines both the number of species in an area and the relative abundance of those species within that area. The result showed that the Asasse Pa beach had higher species richness and species evenness, which resulted in a higher species diversity (Table 3). The higher community parameters at the Asasse Pa beach could be linked to the relatively fewer anthropogenic activities at that place, compared to the Hutchland beach, where anthropogenic activities are more. The anthropogenic activities recorded at the Hutchland beach included the removal of vegetation cover, infrastructure development for commercial activities such as restaurant and recreational facilities, and waste disposal. These activities have resulted in environmental degradation including habitat fragmentation and plastic pollution. Higher species diversity means a higher number of equitably distributed individuals among the species habituating that area. Equitable distribution of resources such as water, nutrients, and light in a fairly stable community is almost certain [50], as shown at the Asasse Pa beach. This indicates that at the Asasse Pa beach, there was equitable utilisation of resources, and this resulted in equitable species distribution as well as coexistence among species, both young recruits and mature ones [50]. This assertion was reflected in the PCA, where minimal variations were observed in the percent variance at the Asasse Pa beach (Table 4), confirming a relatively uniform distribution of the plant species.

The Hutchland beach is a stamping ground in Cape Coast; therefore, human pressures such as stamping and packing of vehicles on the vegetation have resulted in habitat loss and degradation. These, in turn, affected the equitable distribution of species resulting in plant biodiversity loss. The lower species diversity observed at the Hutchland beach was probably due to the inequitable distribution of resources brought about by severe human impacts at that beach. The consequent effects of high human activities such as waste generation from the recreational centre and restaurant tend to favour the establishment and growth of non-shoreline plants [50]. This phenomenon might have resulted in increased competition between shoreline and non-shoreline plants for resources, which might have accounted for the lower species diversity and uneven distribution, as well as high differences in percent variance from the PCA. This may explain why the Hutchland beach recorded more invasive weeds including *Dactyloctenium aegyptium* and *Paspalum vaginatum* as the ecologically important species.

Invasive plant species are recognized as one of the major threats to biodiversity worldwide [56]. In Ghana, the availability and distribution of invasive alien species have been a serious threat to Ghana's biodiversity for many years [57]. Invasive species are regarded as one of the most challenging environmental problems of the 21st century and the second factor causing biodiversity loss [58].

Since species diversity is a widely used tool for measuring the ecological integrity of ecosystems worldwide, the outcome of this study suggests that anthropogenic activities along the shores are having detrimental effects on plant biodiversity along the shorelines of Cape Coast. This is of great concern because species loss can reduce ecosystem functioning and eliminate species that would otherwise improve ecosystem functioning [59]. Moreover, coastal and wetland ecosystems are most vulnerable to invasive threats, whereas the remaining ecosystems show better resilience to invasive threats [60]. Hence, attention must be directed at maintaining plant biodiversity in the coastal areas, to ensure their sustainability.

## 5. Conclusion

In conclusion, it can be said that an attempt was made in this study to inventory plant species along the shoreline of Cape Coast, Ghana, and assess the impacts of human activities on the species composition, as well as on the shoreline ecosystem. From the study, it was clear that human activities along the shoreline have impacted negatively on this ecosystem, comprising the plant composition and distribution. This confirmed the hypothesis that the disturbed area had a lower species diversity due to human activities. There is an urgent need to reduce human activities along the shores to help maintain a stable ecological balance.

Mitigation strategies such as planting coconut trees along the shoreline to restore the coastal habitat, regular clearing of invasive species to ensure the establishment of young recruits of the native species, and the protection of the coastal habitats by having regular beach clean-up will help increase plant biodiversity along the coastline. It is suggested that policymakers, including the Environmental Protection Agency (EPA), the Ministry of Fisheries and Aquaculture Development, and the Cape Coast Metropolitan Assembly enforce regulations on human activities along the coastline. The coastline is regarded as an ecologically sensitive habitat, hence, there must be enforcement of the laws and regulations related to coastal habitats. Continuous and regular monitoring of the activities along the coastline, in addition to effective public awareness and education, will help to maintain healthy coastlines in Ghana. Also, collaboration between policymakers and the coastal inhabitants is very necessary to ensure the long-term sustainability of our coastal habitats. This also calls for an integration of strategic coastal planning and integrated zone management into regional spatial planning for effective coastal protection.

## Figures and Tables

**Table 1:** List of plant species and their taxonomic families.

Families	Species	Habit
Acanthaceae	*Ruellia tuberosa* Linn.	Herb

Aizoaceae	*Trianthema portulacastrum* Linn.	Herb

Amaranthaceae	*Alternanthera pungens* H.B. and K.	Herb
	*Amaranthus spinosus* Linn.	Herb
	*Cyathula prostrata* Linn.	Herb
	*Gomphrena celosioides* Mart.	Herb
	*Pupalia lappacea* Juss.	Herb

Arecaceae	*Cocos nucifera* Linn.	Tree

Asteraceae	*Aspilia africana* (Pers.) C. D. Adams	Herb
	*Lactuca taraxacifolia* (Willd.) Schumach	Herb
	*Synedrella nodiflora* Gaertn. Linn.	Herb

Cactaceae	*Opuntia ficus-indica* (L.) Mill.	Shrub

Caesalpinaceae	*Cassia occidentalis* Linn.	Shrub

Combretaceae	*Terminalia catappa* Linn.	Tree

Commelinaceae	*Aneilema beniniense* (P. Beauv.) Kunth	Herb
	*Commelina benghalensis* Linn.	Herb
	*Commelina diffusa* Burm.f.	Herb

Cucurbitaceae	*Momordica charantia* Linn.	Herb

Cyperaceace	*Cyperus esculentus* Lativum	Herb (sedge)
	*Cyperus rotundus* Linn.	Herb (sedge)
	*Mariscus longibracteatus* Chermezon	Herb (sedge)

Euphorbiaceae	*Euphorbia heterophylla* Linn.	Herb

Mimosaceae	*Schrankia leptocarpa* DC.	Herb

Malvaceae	*Abutilon mauritianum* Mill.	Shrub
	*Malavastrum coromandelianum* Linn.	Herb
	*Sida cordifolia* Linn.	Herb
	*Thespesia populnea* Linn.	Tree

Nyctaginaceae	*Boerhavia diffusa* Linn.	Herb

Papilionaceae	*Baphia nitida* Lodd.	Shrub
	*Canavalia rosea* Sw.	Herb
	*Indigofera hirsuta* Linn.	Herb

Phyllanthaceae	*Phyllanthus amarus* Schumach. and Thonn.	Herb

Poaceae	*Axonopus compressus* Sw.	Herb (grass)
	*Cenchrus biflorus* Roxb.	Herb (grass)
	*Chloris pilosa* Sw.	Herb (grass)
	*Cynodon dactylon* Linn.	Herb (grass)
	*Dactyloctenium aegyptium* (Linn.) P. Beauv.	Herb (grass)
	*Imperata cylindrica* Linn.	Herb (grass)
	*Panicum maximum* Jacq.	Herb (grass)
	*Paspalum vaginatum* Linn.	Herb (grass)
	*Setaria pallide-fusca* (Schumach.) Stapf and C.E. Hubb.	Herb (grass)
	*Sporobolus pyramidalis* P. Beauv.	Herb (grass)

Portulacaceae	*Portulaca oleracea* Linn.	Herb
	*Talinum triangulare* Jacq.	Herb

Rubiaceae	*Diodia scandens* Sw.	Herb
	*Oldenlandia corymbos*a Linn.	Herb

Scrophulariaceae	*Capraria biflora* Linn.	Herb

Solanaceae	*Physalis angulata* Linn.	Herb
	*Solanum lycopersicum* Linn.	Herb

Zygophyllaceae	*Tribulus terrestris* Linn.	Herb

**Table 2:** Ecological parameters of species along the coastline.

Plant species	Frequency (%)	Density (m^−2^)	Abundance (m^−2^)	RF	RD	RA	IVI	Composition (%)
*Abutilon mauritianum*	5	0.6	6	0.88	0.38	1.46	2.72	0.38
*Alternanthera pungens*	5	1.9	19	0.88	1.19	4.64	6.7	1.19
*Amaranthus spinosus*	5	0.7	7	0.88	0.44	1.71	3.02	0.44
*Aspilia africana*	5	0.9	9	0.88	0.56	2.2	3.64	0.56
*Axonopus compressus*	15	4.4	14.67	2.63	2.75	3.58	8.96	2.75
*Baphia nitida*	5	3.2	32	0.88	2	7.81	10.7	2
*Boerhavia diffusa*	10	4	20	1.75	2.5	4.88	9.14	2.5
*Canavalia rosea*	45	13.1	14.56	7.89	8.19	3.55	19.6	8.19
*Capraria biflora*	10	1.2	6	1.75	0.75	1.46	3.97	0.75
*Cenchrus biflorus*	5	0.7	7	0.88	0.44	1.71	3.02	0.44
*Chloris pilosa*	10	3.2	16	1.75	2	3.91	7.66	2
*Commelina benghalensis*	5	0.7	7	0.88	0.44	1.71	3.02	0.44
*Commelina diffusa*	20	3.6	9	3.51	2.25	2.2	7.96	2.25
*Cyathula prostrata*	5	0.5	5	0.88	0.31	1.22	2.41	0.31
*Cynodon dactylon*	25	14.5	29	4.39	9.06	7.08	20.5	9.06
*Cyperus esculentus*	15	3	10	2.63	1.88	2.44	6.95	1.88
*Cyperus rotundus*	35	8.8	12.57	6.14	5.5	3.07	14.7	5.5
*Dactyloctenium aegyptium*	40	30.5	38.13	7.02	19.1	9.31	35.4	19.1
*Diodia scandens*	25	6	12	4.39	3.75	2.93	11.1	3.75
*Euphorbia heterophylla*	5	0.5	5	0.88	0.31	1.22	2.41	0.31
*Gomphrena celosioides*	40	4.6	5.75	7.02	2.88	1.4	11.3	2.88
*Imperata cylindrica*	5	1.2	12	0.88	0.75	2.93	4.56	0.75
*Indigofera hirsuta*	30	5.2	8.67	5.26	3.25	2.12	10.6	3.25
*Lactuca taraxacifolia*	5	0.2	2	0.88	0.13	0.49	1.49	0.13
*Malvastrum coromandelianum*	10	1.1	5.5	1.75	0.69	1.34	3.78	0.69
*Oldenlandia corymbosa*	5	0.4	4	0.88	0.25	0.98	2.1	0.25
*Paspalum vaginatum*	45	18.3	20.33	7.89	11.4	4.96	24.3	11.4
*Phyllanthus amarus*	5	0.9	9	0.88	0.56	2.2	3.64	0.56
*Physalis angulate*	10	1.7	8.5	1.75	1.06	2.08	4.89	1.06
*Pupalia lappacea*	10	0.9	4.5	1.75	0.56	1.1	3.42	0.56
*Ruellia tuberosa*	25	3.3	6.6	4.39	2.06	1.61	8.06	2.06
*Schrankia leptocarpa*	10	0.7	3.5	1.75	0.44	0.85	3.05	0.44
*Setaria pallide-fusca*	5	0.5	5	0.88	0.31	1.22	2.41	0.31
*Spororobolus pyramidalis*	5	0.8	8	0.88	0.5	1.95	3.33	0.5
*Trianthema portulacastrum*	10	1.6	8	1.75	1	1.95	4.71	1
*Tribulus terrestris*	50	16.3	16.3	8.77	10.2	3.98	22.9	10.2

**Table 3:** Ecological community parameters for Asasse Pa and Hutchland beaches.

Parameters	Asasse Pa beach	Hutchland beach
Total number of species	29	25
Species diversity	2.84	2.44
Species evenness	0.84	0.75
Similarity index	0.54	

**Table 4:** Eigen values and their contributions to the correlations.

Principal components	Hutchland beach			Asasse Pa beach		
	Eigen values	% Variance	Cumulative % variance	Eigen values	% Variance	Cumulative % variance
Dim.1	5.6165640	22.466256	22.46626	5.517674	19.705977	19.705977
Dim.2	5.0864637	20.345855	42.81211	4.409201	15.747145	35.45312
Dim.3	4.1236742	16.494697	59.30681	4.266154	15.236265	50.68939
Dim.4	3.6798869	14.719548	74.02636	3.497027	12.489381	63.17877
Dim.5	2.6120959	10.448383	84.47474	2.829861	10.106648	73.28542

**Table 5:** Plant species contributions to the principal components at the Hutchland beach.

Plant species	Principal components				
	Dim.1	Dim.2	Dim.3	Dim.4	Dim.5
*Canavalia rosea*	13.707287	2.20256122	0.038105747	0.01281043	0.3073411
*Indigofera hirsuta*	2.520391	1.72921640	0.012691190	2.00590679	2.1509604
*Diodia scandens*	2.176985	9.51301855	0.996490995	1.83626549	5.9457669
*Boerhavia diffusa*	2.443092	0.08100227	0.121780688	0.12843257	0.5212651
*Paspalum vaginatum*	12.421262	0.70862135	0.009113107	1.21853253	3.7494968
*Cyperus rotundus*	9.013198	2.61003831	0.319548784	0.07049547	3.3558014

**Table 6:** Plant species contributions to the principal components at the Asasse Pa beach.

Plant species	Principal components				
	Dim.1	Dim.2	Dim.3	Dim.4	Dim.5
*Tribulus terrestris*	2.7248285	7.8209746	2.000249	1.5691474	4.593095811
*Ruellia tuberosa*	8.5147856	0.2577918	7.895065	3.5647933	0.322024280
*Canavalia rosea*	1.4595604	0.7487461	4.097613	0.0999524	0.289033179
*Paspalum vaginatum*	0.4534599	8.9252092	2.068040	6.5198438	0.008339793
*Euphorbia heterophylla*	1.7221234	3.8137320	2.915294	14.3518880	1.916900639
*Alternanthera pungens*	1.7221234	3.8137320	2.915294	14.3518880	1.916900639

## Data Availability

All the data that were gathered for this study were analysed, interpreted, and presented within the article.
